# Relationship between orofacial dysfunction and orofacial features, oral function, and eating performance among preschool children

**DOI:** 10.1016/j.jds.2023.06.022

**Published:** 2023-07-05

**Authors:** Mei-Chen Chang, Hsiu-Lin Chen, Shun-Te Huang, Hsiao-Ping Wang, Hsiu-Yueh Liu

**Affiliations:** a Department of Oral Hygiene, College of Dental Medicine, Kaohsiung Medical University, Kaohsiung, Taiwan; b Division of Emergency Medicine and Department Nursing, Kaohsiung Veterans General Hospital, Kaohsiung, Taiwan; c Department of Respiratory Therapy, College of Medicine, Kaohsiung Medical University, Kaohsiung, Taiwan; d Division of Neonatology and Department of Pediatric, Kaohsiung Medical University Hospital, Kaohsiung, Taiwan; e Division of Pediatric Dentistry and Special Needs Dentistry, Department of Dentistry, Kaohsiung Medical University Hospital, Kaohsiung, Taiwan; f Division of Pediatric Medicine and Department of Neonatology, Kaohsiung Veterans General Hospital, Taiwan; g Department of Medical Research, Kaohsiung Medical University Hospital, Kaohsiung, Taiwan

**Keywords:** Chewing, NOT-S, Oral habits, Preterm, Swallowing

## Abstract

**Background/purpose:**

Orofacial (OF) development is influenced by multiple factors. This study aimed to explore the relationship between OF dysfunction (OFD) and OF features, oral function, and eating performance among preschool children.

**Materials and methods:**

There were 243 preschool children and their parents who participated in this cross-sectional study. Participant demographic information and eating performance were obtained from questionnaires completed by their mothers. OF features and functions were assessed using oral examinations. OFD assessments were performed using Nordic Orofacial Test–Screening (NOT-S).

**Results:**

Approximately 80% of participants had at least one domain of NOT-S affected. The main OFD in a structured interview was chewing and swallowing (64.61%). Dysarthria (40.38%), weak bite force (53.85%), inability to effectively chew (45.19%), and taking longer than 30 min to eat meals (75.00%) were significantly more prevalent among participants with OFD than among those without OFD (all *P* < 0.05). Also, compared with participants born full-term, those born prematurely and who had OFD had higher rates of V-shaped dental arch (42.11%), high-arched palate (31.58%), small mouth opening capacity (7.89%), dysarthria (65.79%), preference to eating soft-textured food (42.11%), and weak cough strength (21.05%). Taking longer than 30 min to eat meals (adjusted odds ratio (AOR = 8.87, *P* < 0.001) and not effectively chewing food (AOR = 8.81, *P* < 0.001) were significantly associated with OFD.

**Conclusion:**

Chewing and swallowing and habits are common among preschool children and associated with OFD. OFD is associated with OF features, and presented in oral function and eating performance.

## Introduction

The preterm birth (PB) rate in Taiwan was 8.2% in 2001. In 2011 and 2021, this rate was 9.1% and 10.6%, respectively,^1^, ^2^, ^3^ representing a nearly 30% increase over a 10-year period. PBs require more-intensive neonatal care and are generally accompanied by additional complications, such as respiratory distress syndrome, bronchopulmonary dysplasia, epileptic seizures, cerebral palsy, infections, and feeding difficulties.^4^, ^5^, ^6^, ^7^, ^8^, ^9^ The immediate consequences and long-term effects of PBs on the physical and psychological development of children born prematurely are topics that have received a great deal of attention. Physical and mental development during the early childhood stage, including the development of orofacial (OF) structures and functions, is markedly delayed among children born prematurely.^10^

OF structure affects the three major functions of breathing, eating, and speaking and is also related to chewing, swallowing, drooling, habits (such as finger biting, finger sucking, tongue thrusting, lip biting, or lip sucking etc.), occlusion, and oral health.^11^, ^12^, ^13^, ^14^ The human nervous system matures within the first few years after birth, and OF structure grows rapidly during this time.^15^ Children born prematurely may have OF dysfunction (OFD) due to an immature nervous system. OFD manifests as oromandibular system sensorimotor dysfunction, increased muscle hypotonia, or inability to effectively swallow.^8^^,^^10^^,^^16^, ^17^, ^18^ Preterm infants with OFD are at a high risk of feeding difficulties, especially if they have physiological symptoms, and must receive selective/restricted feeding.^19^

OF development is a complex process influenced by factors such as PB, negative feeding experiences, and habits.^10^^,^^20^, ^21^, ^22^ These factors may result in changes to oral morphology and oral function that affect eating, drinking, and speaking difficulties and may persist into childhood.^19^^,^^23^ Studies on OF developmental problems have generally focused on the association between birth weight or/and gestational age with OF development. A study suggested assessing OF features and subsequent functional development problems.^8^ Accordingly, the present study examined the correlations between OFD, OF features, oral function, and eating performance among 3–5-year-old preschool children. Additionally, this study explored whether the factors influencing OFD varied between the PB and full-term birth (FTB) groups.

## Material and methods

### Study participants

This observational, cross-sectional study recruited 243 preschool children aged 3–5 years old and their mothers (i.e., the primary caregivers) recruited from two medical centers in southern Taiwan. Preschool children with a systemic disease, mental disability, congenital craniofacial abnormalities, or cerebral palsy were excluded.

## Ethical approval

This study was approved by the human experiment and ethics committees of two medical centers (protocol number: KMUHIRB-20140107 and VGHKS15-CT3-11). Prior to performing the oral examinations and oral function assessments, the principal investigators explained the purpose and content of the study to the study participants in detail. All parents or guardians signed a written informed consent form after agreeing to participate in the study with their children.

### Questionnaire and oral examination

We used a structured questionnaire to collect the demographic information of the participants and their chewing and swallowing performance. Gestational age (GA) is a common criterion and is widely used to assess fetal maturity when infants are born. The participants were classified into 2 groups: PB (GA <37 weeks) and FTB (GA ≥37 weeks).^24^ Participants were further divided into subgroups according to body mass index values recommended by the Taiwan Health Promotion Administration (HPA) in 2018.^25^

We performed an expert validity and reliability test on the questionnaire before data collection. In terms of expert validity, six experts with relevant knowledge and experience conducted a validity test on the applicability of the questionnaire. The content validity index of the questionnaire ranged between 0.88 and 1.00, and the overall validity was 0.96. After the questionnaire content validity passed expert review, we conducted a pretest interview with five mothers of preschool children. A week later, we interviewed the five mothers again using the same questionnaire, obtaining a retest reliability of 1.00. The mothers completed the questionnaire prior to OF functional assessment. An independent senior pediatric nurse who had received dental training assisted all mothers in completing the questionnaire.

After the mothers completed the questionnaire, we performed oral examinations on all participants to obtain information on OF features and perform oral function assessments. The content of the OF and eating performance assessment questionnaire was designed to take into account the factors that affect chewing and swallowing performance.^26^ Swallowing function was evaluated using the repetitive saliva swallowing test (RSST) in which the children were asked to swallow saliva as many times as possible for 30 s.^27^ OFD was assessed using the Nordic Orofacial Test–Screening (NOT-S) instrument and the assessment took 5–7 min. The NOT-S has been proven to be valid and reliable in identifying OFD in children aged older than 3 years and has a screening sensitivity and specificity of 0.96 and 0.63, respectively.^11^ The mothers assisted their children in answering the interview questions during the assessments.

NOT-S, which contains a structured interview and a clinical examination, has 12 domains.^11^ The structured interview consists of six domains including sensory function, breathing, habits, chewing and swallowing, drooling, and dryness of the mouth. The clinical examination consists of six domains that measure face at rest, and tasks regarding, nose breathing, facial expression, masticatory muscle and jaw function, oral motor function, and speech. Each domain has 1 to 5 options. If the answer to any one of the questions or if the performance of any one of the functions met the dysfunction criteria, the domains were recorded as a “yes.” For each assessment domain, one point was given if one or more answers were “yes.” The total NOT-S score ranged from 0 to 12 points; the higher total score, the higher the number of OF dysfunction. A NOT-S score of two was set as the cutoff point. A score of two or more indicated OFD.

### Statistical analysis

IBM SPSS 20.0 (IBM, Armonk, NY, USA) was used for statistical analysis. Chi-square and Fisher's exact tests were conducted to compare the percentage differences between the two groups, and a *t*-test was performed to analyze differences in continuous data between the two groups. Variables affecting OFD were explored using univariate and multivariate logistic regression analyses. The significance level for all statistical tests was set at *P* < 0.05, and the confidence interval (CI) was set at 95%.

## Results

The number of participants born prematurely was evenly distributed across all ages, whereas the number of participants born full-term was significantly less in the 3-year age group (*P* = 0.035; Table 1). The percentage of participants with at least one OF domain affected was lower in the PB group than in the FTB group (72.55% vs. 85.11%). For both groups, the most commonly reported OF functional difficulties were chewing and swallowing. Approximately 59.80% of participants in the PB group and 68.09% of participants in the FTB group had chewing and swallowing difficulties. The percentage of participants with habits in the FTB group (53.09%) was 1.5 times higher than that of participants in the PB group (35.29%; *P* = 0.004; Table 2), and the difference was significant. For the PB group, NOT-S scores decreased with age, decreasing from 1.34 for participants aged 3 years to 1.24 for participants aged 5 years. For the FTB group, NOT-S scores increased with age, increasing from 1.34 for participants aged 3 years to 1.65 for participants aged 5 years (Table 3).

**Table 1:** Participant characteristics.

Variable	N	(%)	Preterm		Full-term		*P*-value
			n	%	n	%	
Total	243		102	41.98	141	58.02	
Gender							
Male	117	48.15	47	46.08	70	49.65	0.605
Female	126	51.85	55	53.92	71	50.35	
Age^a^	4.14	0.82	4.06	0.90	4.19	0.76	0.229
3 years old	64	26.34	35	34.31	29	20.57	0.035
4 years old	86	35.39	29	28.43	57	40.43	
5 years old	93	38.27	38	37.25	55	39.01	
Gestational age at birth^a^	35.84	4.16	31.84	3.46	38.74	1.05	<0.001
Birth weight^a^	2500.09	897.09	1640.27	671.78	3122.09	370.48	<0.001
Body Mass Index Level							
Underweight	39	16.05	18	17.65	21	14.89	0.032
Healthy weight	148	60.91	69	67.65	79	56.03	
Overweight or obesity	56	23.05	15	14.71	41	29.08	
NOT-S score (0–4)^a^	1.41	1.03	1.28	1.08	1.50	0.98	0.102
Interview score (0–3)^a^	1.24	0.87	1.07	0.87	1.37	0.85	0.008
Examination score (0–1)^a^	0.17	0.38	0.22	0.41	0.13	0.34	0.097

a Presented as mean and standard deviation. NOT-S: Nordic Orofacial Test–Screening.

**Table 2:** Distribution of NOT-S domains among preschool children.

Variable	All		Preterm		Full-term		*P*-value
	n	%	n	%	n	%	
NOT-S score							
0	49	20.16	28	27.45	21	14.89	0.072
1	90	37.04	36	35.29	54	38.30	
2	64	26.34	21	20.59	43	30.50	
≥3	40	16.46	17	16.67	23	16.31	
NOT-S interview							
Breathing-snoring while sleeping	31	12.76	12	11.76	19	13.48	0.846
Habits	112	46.09	36	35.29	76	53.90	0.004
Bite nail, or suck fingers, or other objects	76	31.28	24	23.53	52	36.88	0.035
Bite teeth together hard or grind teeth	53	21.81	15	14.71	38	26.95	0.027
Suck or bite lips, tongue, or cheeks	29	11.93	11	10.78	18	12.77	0.692
Chewing and swallowing	157	64.61	61	59.80	96	68.09	0.221
Take 30 min or more to eat a main meal	125	51.44	48	47.06	77	54.61	0.298
Swallow large bites without chewing	66	27.16	23	22.55	43	30.50	0.190
Drooling	2	0.82	0	0.00	2	1.42	0.511
NOT-S examination							
Speech	41	16.87	22	21.57	19	13.48	0.118

NOT-S: Nordic Orofacial Test–Screening.

**Table 3:** NOT-S score among preschool children.

Variable	All		*P*-value	Preterm		*P*-value	Full-term		*P*-value
	Mean	SD		Mean	SD		Mean	SD	
NOT-S score (0–4)	1.41	1.03		1.28	1.08		1.50	0.98	0.102
Interview score (0–3)	1.24	0.87		1.07	0.87		1.37	0.85	0.008
Examination score (0–1)	0.17	0.38		0.22	0.41		0.13	0.34	0.097
Gender									
Male	1.49	1.07	0.271	1.32	1.16	0.766	1.60	1.00	0.249
Female	1.34	0.99		1.25	1.02		1.41	0.96	
Age									
3 years old	1.34	1.01	0.673	1.34	1.03	0.917	1.34	1.01	0.318
4 years old	1.38	1.05		1.28	1.25		1.44	0.95	
5 years old	1.48	1.03		1.24	1.02		1.65	1.00	
Gestational age at birth									
24–27 weeks	1.60	1.35	0.231	1.60	1.35	0.485			
28–31 weeks	1.34	1.09		1.34	1.09				
32–36 weeks	1.18	1.03		1.18	1.03				
≥37 weeks	1.50	0.98					1.50	0.98	
Birth weight									
<1000 g	1.60	0.99	0.267	1.60	0.99	0.512			
1000–1499 g	1.33	1.16		1.33	1.16				
1500–2499 g	1.13	0.91		1.08	1.02		1.25	0.46	0.455
≥2500 g	1.48	1.01		1.18	1.07		1.52	1.00	
Body Mass Index Level									
Underweight	1.59	0.82	0.245	1.44	0.86	0.776	1.71	0.78	0.231
Healthy weight	1.32	1.07		1.26	1.15		1.38	1.00	
Overweight or obesity	1.52	1.04		1.20	1.08		1.63	1.02	

SD: Standard Deviation; NOT-S: Nordic Orofacial Test–Screening.

In terms of oral function and eating performance, participants with OFD performed worse than those without OFD. Dysarthria (40.38%), weak bite force (53.85%), inability to effectively chew food (45.19%), and taking longer than 30 min to eat meals (75.00%) were significantly more prevalent among participants with OFD than among those without OFD (all *P* < 0.005; Table 4). The participants with OFD, V-shaped dental arch (42.11%), high-arched palate (31.58%), small mouth opening capacity (7.89%), dysarthria (65.79%), eating soft-textured food (42.11%), and weak cough strength (21.05%) were significantly more prevalent in the PB group than in the FTB group (all *P* < 0.005; Table 5).

**Table 4:** Orofacial features, oral function, and eating performance by orofacial dysfunction of participants.

Variable	N	(%)	OFD-free		OFD		*P*-value
			n	%	n	%	
Total	243		139		104		
Dental arch form							
U shaped	191	78.60	115	60.21	76	39.79	0.082
V shaped	52	21.40	24	17.27	28	26.92	
High-arched palate							
Absent	206	84.77	122	87.77	84	80.77	0.151
Present	37	15.23	17	12.23	20	19.23	
Open bite							
Absent	232	95.47	136	97.84	96	92.31	0.059
Present	11	4.53	3	2.16	8	7.69	
Dysarthria							
Absent	165	67.90	103	74.10	62	59.62	0.019
Present	78	32.10	36	25.90	42	40.38	
Maximal mouth opening capacity							
21–30 mm	7	2.88	4	2.88	3	2.88	0.998
>31 mm	236	97.12	135	97.12	101	97.12	
Dental caries							
Free	162	66.67	100	71.94	62	59.62	0.054
Active	81	33.33	39	28.06	42	40.38	
Repetitive Saliva Swallowing Test							
≥3 times	160	65.84	93	66.91	67	64.42	0.785
<3 times	83	34.16	46	33.09	37	35.58	
Bite force							
Strong	154	63.37	106	76.26	48	46.15	<0.001
Weak	89	36.63	33	23.74	56	53.85	
Chewing food							
Effectively	177	72.84	120	86.33	57	54.81	<0.001
Ineffectively	66	27.16	19	13.67	47	45.19	
Food textures							
Raw foods	196	80.66	118	84.89	78	75.00	0.070
Soft-textured foods	47	19.34	21	15.11	26	25.00	
Coughing during a meal							
Absent	232	95.47	134	96.40	98	94.23	0.536
Present	11	4.53	5	3.60	6	5.77	
Cough strength							
Strong	228	93.83	132	94.96	96	92.31	0.429
Weak	15	6.17	7	5.04	8	7.69	
Eating duration							
≤30 min	118	48.56	92	66.19	26	25.00	<0.001
＞30 min	125	51.44	47	33.81	78	75.00	

OFD: Orofacial dysfunction.

**Table 5:** Orofacial dysfunction among preschool children.

Variable	Preterm		Full-term		*P*-value
	n	%	n	%	
Total	38	37.25	66	46.81	
Dental arch form					
U shaped	22	57.89	54	81.82	0.012
V shaped	16	42.11	12	18.18	
High-arched palate					
Absent	26	68.42	58	87.88	0.021
Present	12	31.58	8	12.12	
Open bite					
Absent	34	89.47	62	93.94	0.459
Present	4	10.53	4	6.06	
Dysarthria					
Absent	13	34.21	49	74.24	<0.001
Present	25	65.79	17	25.76	
Maximal mouth opening capacity					
21–30 mm	3	7.89	0	0.00	0.046
>31 mm	35	92.11	66	100.00	
Dental caries					
Free	23	60.53	39	59.09	0.886
Active	15	39.47	27	40.91	
Repetitive Saliva Swallowing Test					
≥3 times	24	63.16	43	65.15	0.835
<3 times	14	36.84	23	34.85	
Bite force					
Strong	16	42.11	31	46.97	0.851
Weak	22	57.89	35	53.03	
Chewing food					
Effectively	22	57.89	56	84.85	0.686
Ineffectively	16	42.11	10	15.15	
Food textures					
Raw foods	24	63.16	54	81.82	0.004
Soft-textured foods	16	42.11	10	15.15	
Coughing during a meal					
Absent	37	97.37	61	92.42	0.412
Present	1	2.63	5	7.58	
Cough strength					
Strong	30	78.95	66	100.00	<0.001
Weak	8	21.05	0	0.00	
Eating duration					
<30 min	13	34.21	13	19.70	0.108
≥30 min	25	65.79	53	80.30	

Associations between OFD risk and the related variables assessed by univariate logistic regression analysis are presented in Table 6. No significant association was detected between OFD risk and preterm birth. Further, the multivariate logistic regression analysis demonstrated that dysarthria (AOR = 4.90, 95% CI: 210–12.34, *P* < 0.001), not effectively chewing food (AOR = 8.81, 95% CI: 4.19–19.83, *P* < 0.001) and taking longer than 30 min to eat meals (AOR = 8.87, 95% CI: 4.53–18.52, *P* < 0.001) were considered as independent predictive variables for OFD (Table 6).

**Table 6:** Factors of orofacial dysfunction among preschool children.

Variables and coding	COR^a^	95% CI		*P*-value	AOR^b^	95% CI		*P*-value
		Lower	Upper			Lower	Upper	
All participants								
Male (vs Female)	1.30	0.78	2.17	0.309	1.47	0.79	2.76	0.221
Age (Years old)	1.67	0.66	4.28	0.279	2.09	0.66	6.70	0.212
Dysarthria (vs. Absent)	1.94	1.13	3.36	0.017	2.61	1.36	5.12	0.005
Dental caries-active (vs. Dental caries-free)	1.74	1.01	2.99	0.045				
Bite force weak (vs. Strong)	3.75	2.18	6.55	<0.001				
Ineffectively chewing food well (vs. Effectively)	5.21	2.84	9.86	<0.001	8.89	4.28	19.80	<.0001
Eating duration ≥30 min (vs. < 30 min)	5.69	3.27	10.15	<0.001	8.64	4.48	17.70	<.0001

CI: confidence interval. a COR: crude odds ratio. Data analysis by univariate logistic regression model: dependent variable was NOT-S score of 2 or more. Adjusted for participants' sex and age. b AOR: adjusted odds ratio. Data analysis by multiple logistic regression model. Independent variables were analyzed in the multivariate logistic regression model only if they were found to be significantly correlated with OFD in the univariate logistic regression analysis. Logistic regression model: dependent variable was NOT-S score of 2 or more. Adjusted for participants' sex and age.

## Discussion

The most common OF problems were chewing and swallowing, followed by habits. The FTB group exceeded the PB group in percentages in all OFD domains except for speech. Oral dysfunction is associated with abnormal oral features and the weakening of daily oral function and eating performance. In the present study, we did not have sufficient evidence to support that PB is a risk factor for OFD, suggesting that OFD is more likely the result of acquired habits.

Studies on the early and long-term effects of PB on the development of OF structure in children have had inconsistent findings.^10^ Oral motor functions, as is the case with most physical motor functions, develop gradually during preschool years. This phenomenon can be corroborated by the fact that the total NOT-S score shows a slight downward trend with an increase in gestational age at birth and birth weight among participants in the PB group. However, the relationship between NOT-S score and age in the present study differed from that in previous studies. The total NOT-S score in the FTB group increased with age, and the percentage of participants with habits in the FTB group also increased from 41.38% at age 3–60.00% at age 5. This suggests that acquired habits are a factor influencing oral function and chewing and swallowing performance in the FTB group.

Snoring is a sign of breathing disorder and is common among preschool children.^28^^,^^29^ In the FTB group, 13.48% of participants snored while sleeping, and 52.63% of them were obese. In contrast, in the PB group 11.76% snored, but this was related to oral habits. Long-term mouth breathing decreases oral muscle tension, restricts the development of the palate and OF structures, and narrows the arched palate, resulting in disorderly breathing patterns during sleep and snoring.^30^^,^^31^ This result was verified by the PB group results of this study. That is, compared with the FTB group, the percentages of participants in the PB group with V-shaped dental arch and high-arched palate (42.11% and 31.58%, respectively) were both 2 times higher than those of participants in the FTB group (18.18% and 12.12%, respectively).

Oral habits are abnormal habits.^12^^,^^28^ The results of this study showed that 53.90% of the FTB group and 35.29% of the PB group had at least one oral habit. Consistent with previous studies, finger sucking, and nail biting are the most common behavior in childhood.^14^^,^^32^^,^^33^ Due to difficulty noticing other oral habits, such as sucking or biting lips, tongue, or inner cheek habits are difficult to observe in children and showed a lower prevalence. Continuing to engage in nonnutritive sucking habits after the age of 2 or 3 years greatly increases the likelihood of anterior open bite and posterior malocclusion.^10^^,^^32^, ^34^, ^35^, ^36^ Additionally, we found that the percentage of participants with habits gradually increased from 42.19% at age 3–51.61% at age 5. Over time, oral habits significantly affect lip strength (i.e., lack of lip strength) and craniofacial development (e.g., greater overjet, greater palatal depth, narrower maxillary arch width).^33^ Preschool children with habits and muscle functions that are not eliminated or corrected early may require orthodontic treatment.^36^

In the present study, chewing and swallowing, as recorded in the NOT-S scale, were the most common OF problems, consistent with the findings of previous studies.^12^^,^^15^^,^^28^ Through the RSST, no difference was found in the proportion of swallowing function between the OFD participants and OFD-free participants or between PB and FTB groups. We found a weak cough strength in the PB group and this may be a negative effect of placement of NG tubes during hospitalization after birth.^26^ We found that not effectively chewing food, small mouth opening capacity, and eating soft textured food were more prevalent in the PB group than in the FTB group. Small mouth opening capacity, weak bite force, and dental caries were associated with OFD and reflected in chewing performance. Preschool children may choose soft textured food to compensate for being unable to effectively chew or repeatedly adjust their chewing behavior to chew the food given to them.^37^ Children born prematurely who have sucking and chewing difficulties are often unaware that their mouths are full of food.^38^^,^^39^ Chewing without swallowing was more prevalent among participants in the FTB group than among participants in the PB group, indicating that chewing and swallowing problems may be the result of habits developed over time.

Speech problems were 2.5 times more prevalent among participants with OFD in the PB group than in the FTB group. This is in line with previous studies that indicated children born before 30 weeks of gestation had lower language skills than those born at term.^22^ We found that dysarthria in the FTB group was 21.05%, 36.84%, and 42.11% for participants aged 3, 4, and 5 years, respectively. In a study by Gustavsson et al.,^12^ 22.5% of children aged 5–7 years had a speech impairment, and less than 10% of children aged 3–4 years had a speech impairment, similar to our findings. During normal development, children begin to master speech and grammar by the age of 5 years. Following more syllable repetition and rigorous pronunciation practice, the demand for speech clarity also increases. Consequently, speech impairment becomes more noticeable with age.^12^^,^^15^^,^^28^

This study has several limitations. First, we were unable to objectively observe the differences in oral function between the preterm and full-term groups to verify the extent of their oral function damage. NOT-S has been used as a screening instrument for oral function since 2005. In recent years, other instruments for objectively measuring oral function have been developed. We suggest that future studies perform swallowing and chewing measurements in addition to using NOT-S to accurately measure oral function. Second, we did not investigate whether participants had diseases or respiratory problems, these may have been confounding factors. Third, the effect of dental caries on chewing and swallowing dysfunction has not been demonstrated in the literature. More research is needed to validate our findings.

Chewing and swallowing are the most common OF problems among preschool children. OFD was more prevalent among preschool children born full-term than among preschool children born prematurely. In the PB group, the second common OF problem was speech, and in the FTB group, the second common OF problem was habits. OFD among preschool children can be identified by observing their eating performance and oral characteristics. The effect of habits on OF development among preschool children is an emerging issue worthy of further observation and discussion.

## Declaration of competing interest

The authors have no conflicts of interest relevant to this article.
